# Rontgen Ray Diagnosis and Therapy

**Published:** 1904-09

**Authors:** 


Rontgen Ray Diagnosis and Therapy. By Carl Beck, M. D., Professor of Surgery in the New York Post-Graduate Medical School and Hospital; Visiting Surgeon to Saint Mark's Hospital and the German Poliklinik. Octavo, pp. 479, with 322 illustrations. New York and London: D. Appleton & Co. 1904. (Price, $4.00.)

This is a well arranged and useful book professedly for beginners, and is designed to afford the means of acquiring the necessary training in *x*-ray diagnosis and therapeutics. It is divided into three sections,—the first is wholly technical in character—special attention being given to apparatus, Röntgen technic, fluoroscopy, skiography and examination of the patient.

The second is clinical in its contribution with special stress laid upon the importance of a thorough knowledge of the anatomy and pathology of the various regions of the body, so that the part examined may be properly placed and the result correctly interpreted, while the third section is devoted to the effects of the Röntgen rays, which is well worth perusal, as the author presents the numerous cases in detail, thus rendering his report more valuable. The book is well written and thoroughly illustrated. The photographic reproductions are distinct and deserve consideration.

